# Comparison of image quality of 3D ultrasound: motorized acquisition versus freehand navigated acquisition, a phantom study

**DOI:** 10.1007/s11548-023-02934-x

**Published:** 2023-05-27

**Authors:** N. M. Bekedam, L. H. E. Karssemakers, M. J. A. van Alphen, R. L. P. van Veen, L. E. Smeele, M. B. Karakullukcu

**Affiliations:** 1 Department of Head and Neck Surgery and Oncology, Netherlands Cancer Institute, Antoni van Leeuwenhoek, Plesmanlaan 121, 1066 CX Amsterdam, The Netherlands; 2 Department of Head and Neck Surgery and Oncology, Verwelius 3D Lab, Netherlands Cancer Institute, Antoni van Leeuwenhoek, Amsterdam, The Netherlands; 3 Academic Centre of Dentistry Amsterdam, Vrije Universiteit, Gustav Mahlerlaan 3004, 1081 LA Amsterdam, The Netherlands

**Keywords:** EM tracking, Freehand, Image quality, Motorized, Three-dimensional, Ultrasound

## Abstract

**Purpose:**

Intra-operative assessment of resection margins during oncological surgery is a field that needs improvement. Ultrasound (US) shows the potential to fulfill this need, but this imaging technique is highly operator-dependent. A 3D US image of the whole specimen may remedy the operator dependence. This study aims to compare and evaluate the image quality of 3D US between freehand acquisition (FA) and motorized acquisition (MA).

**Methods:**

Multiple 3D US volumes of a commercial phantom were acquired in motorized and freehand fashion. FA images were collected with electromagnetic navigation. An integrated algorithm reconstructed the FA images. MA images were stacked into a 3D volume. The image quality is evaluated following the metrics: contrast resolution, axial and elevation resolution, axial and elevation distance calibration, stability, inter-operator variability, and intra-operator variability. A linear mixed model determined statistical differences between FA and MA for these metrics.

**Results:**

The MA results in a statistically significant lower error of axial distance calibration (*p* < 0.0001) and higher stability (*p* < 0.0001) than FA. On the other hand, the FA has a better elevation resolution (*p* < 0.003) than the MA.

**Conclusion:**

MA results in better image quality of 3D US than the FA method based on axial distance calibration, stability, and variability. This study suggests acquiring 3D US volumes for intra-operative *ex vivo* margin assessment in a motorized fashion.

## Introduction

Tongue squamous cell carcinoma (TSCC) is treated by surgical resection of the tumor, including a margin to ensure no tumor cells are left. Widely accepted guidelines categorize resection margins as positive (< 1 mm), close (1–5 mm), and negative (> 5 mm) [1]. Close and positive margins are associated with lower survival and higher recurrence ratios [2, 3] and therefore indicate adjuvant therapy as re-resection or (chemo)radiotherapy (CRT). The consequences of adjuvant therapy are reduced Quality of Life (QoL) and higher treatment costs. Reported numbers show a close or positive margin of up to 85% of the cases [4, 5]. These numbers substantiate the need for intra-operative assessment of the margins to reduce these numbers and, consequently, to reduce the need for adjuvant therapy.

Several techniques are available for intra-operative assessment of the margins. Frozen section histopathology (FSH) is one of the most commonly used. However, a recent study shows that FSH is unreliable and suggests that FSH should not be used [6]. Brouwer de Koning et al. report about hyperspectral diffuse reflection imaging (HIS) [7]. HIS is performed on the specimen’s cross-sections, permanently disrupting tissue and losing anatomical orientation. Several studies report promising results of intra-operative margin assessment with ultrasound (US). Tabarichi et al. report on several intra-operative US studies to achieve adequate deep margins following pathological analysis [8]. Brouwer de Koning et al. show a significant relationship between margins measured by *ex vivo* US and histopathology, with a mean absolute difference of 1.1 (0.9) mm between the measurements [9]. De Koning et al. demonstrate that intra-oral US guidance of the resection with *ex vivo* margin assessment reduces the number of positive margins by three times [10]. A reported drawback is the moderate identification of close margins by the *ex vivo* US since the immediate re-resection and markers indicating the close margin on the specimen are misplaced compared to histopathological analysis. The 2D aspect of *ex vivo* US could explain the reason for misplacement. The acquisition of the US image to be assessed highly depends on the operator [11] and is difficult to reproduce.

A previous study from our center demonstrates the capability of assessing the margins of the entire specimen with three-dimensional (3D) US [12]. Electromagnetic (EM) tracking of the probe enables the acquisition of multiple US images and reconstruction into a 3D US volume. This technique, also called freehand acquisition (FA), allows for assessing the margins of the entire specimen instead of a single slice selected by the operator. Additionally, this method does not require contact with the tissue because the specimen is submerged in saline to prevent tissue deformation, nor does it slice the specimen to maintain anatomical orientation.

Although FA provides the operator to move in all directions and angles, the human tremor is still recognizable in the 3D US volume leading to incorrect shape visualization. Besides, the algorithm to reconstruct 2D images into a 3D volume uses interpolation. Targets with a minimal difference in gray value compared to the surroundings, such as TSCC, are hard to differentiate as the gradient of the tumor borders slightly vanishes by the interpolation of data. A stabilized acquisition method ensuring no tremor [13] and controlling the elevation step size by motor could overcome these limitations. Consequentially, the stabilized acquisition enables the reconstruction of images into a 3D volume by stacking without interpolation. This way, the US system’s original image quality is maintained.

This study aims to compare and evaluate the image quality of 3D US volume between motorized acquisition (MA) and EM-navigated FA, as described previously [12]. The image quality is evaluated following contrast resolution, axial and elevation resolution, axial and elevation distance calibration, stability*,* and variability in acquisitions conducted on a commercial phantom.

## Method

### Materials and setup

In this study, a US system (BK5000, software version: 5.148.18234.26, license: OEM interface, BKMedical, Denmark) with an intra-operative transducer (T-shape, BKMedical, Denmark) performed the acquisitions. The transducer frequency was set to ten MHz, and the depth was adjusted so the imaging targets were in the field of view. This means five cm depth for the distance calibration in elevation direction and 4 cm depth for the other experiments. The auto-focus mode automatically adjusted the focus depth. All experiments were conducted using a commercial phantom, including horizontal and vertical distance calibration filaments and contrast cylinders (040GSE, CIRS, Virginia, USA, URL: https://www.cirsinc.com/products/ultrasound/zerdine-hydrogel/multi-purpose-multi-tisse-ultrasound-phantom/, see Appendix A). A water well (one cm deep) was attached to the phantom to mimic the clinical setting of intra-operative *ex vivo* margin assessment of a submerged specimen.

**Fig. 1:**
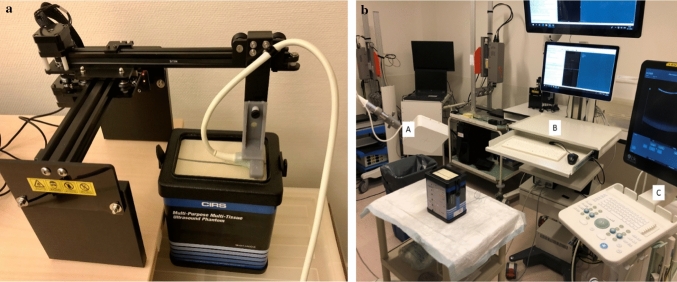
**a** (left): The motorized 3D US system. The black rails with dual stepper motor enable movement in two directions. The 3D-printed clip in gray attaches the US transducer to the rails. The US transducer’s cable is perpendicular to its imaging plane. In the photograph, the imaging plane is vertical through the commercial phantom. **b** (right): The EM-navigated 3D US system. Part A is the EM field generator, part B is the EM sensor interface unit and the system control unit, and part C is the US system. The US system is required in the motorized 3D US system as well

Applying an electromagnetic (EM) tracking system (Aurora, Toolbox version: 5.002.022, NDI, Canada) enables FA (Fig. 1b). Nijkamp et al. assess this system in-house and conclude that within 30 cm of the field generator the tracking accuracy is in the order of 1 mm and 1° [14]. This EM system in our study consists of a field generator (Planar 20–20, NDI, Canada) that creates an EM field in which the phantom was placed. A 6DOF sensor (NDI Technical specifications: position RMS: 0.48 mm, 95%CI: 0,88 mm; orientation RMS: 0.30°, 95%CI: 0.48°; frequency: 800 Hz; measurement rate: 40 Hz) clipped to the US transducer in a 3D-printed mold (the CAD file is available upon request) collects the position and orientation data from the US transducer. CustusX, an image-guided therapy platform [15], enables real-time data acquisition from the US and EM tracking systems to reconstruct these data into a 3D US volume. Probe calibration for position tracking between the sensor and the US image was performed following the method of Bø et al. [16]. In this method, a robot moves a submerged sphere through the US image, followed by segmentation of the sphere in the images. Together with the tracking information the calibration matrix could be computed.

The MA was performed by applying a rails system moved by a stepper motor (motion control accuracy: 0.01 mm) (Master 2s, NEJE, China) and controlled by communicating converted strings of G-code (computer language for automatic machinery) into bytes through a serial connection. A 3D-printed clip attaches the US transducer to the rails, as shown in Fig. 1a. The SciKit-SurgeryBK python library (https://github.com/SciKit-Surgery/scikit-surgerybk; Version: 0.18) enables communication from the workstation to the US system [17].

Image analysis was done using 3DSlicer (RRID: SCR_005619) [18], and statistical analysis was performed in SPSS (Version 27, IBM Corp., New York, USA, RRID: SCR_002865) [19] and Microsoft Excel (2016, Microsoft Corp., RRID: SCR_016137).

### Data acquisition

For FA, probe movement is extracted from tracking and imaging data, and then, the correlation is used to find a shift between the movement data streams with the built-in Temporal Calibration function in CustusX (https://www.custusx.org/uploads/developer_doc/nightly/classcx_1_1_temporal_calibration.html) before the FA took place. The operator holds the transducer below the water surface without touching the phantom. Once the acquisition starts, the operator moves the transducer in one sweep along the phantom’s elevation direction (Z, Fig. 3) and acquires US images in real time. This function finds the shift between the image timestamp and the tracking timestamps.

During MA, the US transducer is attached to the rails and placed above the phantom, with the transducer below the water surface not touching the phantom. The range of motion of the transducer during MA is more limited than during FA, as the transducer’s cable touches the phantom’s water well earlier as the transducer pose cannot be adjusted. As shown in Table 1, the rail system requires several variables as input as the rails system enables these possibilities: step or continuous movement, velocity or step size, and compound imaging. Compound imaging is explained below. The rail system can continuously move the transducer. Step size is estimated with the velocity as input and the known frames per second (FPS) in which the US images are continuously acquired. A stepwise movement of the transducer is possible by the actual step size as input. A single US image is acquired, followed by a single transducer step. After each step, the system waits for 0.5 s to move the transducer to the following location physically and to ensure the transducer is entirely still. Then, the next US image is acquired. The unmoving transducer provides the possibility of compound imaging. Compound imaging is acquiring multiple images of the exact location and then averaging pixel intensities to reduce noise. The US system acquired five US images when applying compound imaging.

**Table 1:** The variables set for each experiment

Experiment number	Operator	Acquisition method	Step size in mm	Movement	Compounding
1	A	Freehand	–	Freehand	–
2	B	Freehand	–	Freehand	–
3	A	Motorized	1.0	Stepwise	False
4	A	Motorized	0.5	Stepwise	False
5	A	Motorized	0.5	Stepwise	True
6	B	Motorized	0.5	Stepwise	False
7	A	Motorized	0.2	Stepwise	False
8	A	Motorized	0.1	Stepwise	False
9	A	Motorized	1.0	Continuous	False
10	A	Motorized	0.5	Continuous	False
11	B	Motorized	0.5	Continuous	False
12	A	Motorized	0.2	Continuous	False
13	A	Motorized	0.1	Continuous	False

### Volume reconstruction

The pixel nearest-neighbor (PNN) algorithm in CustusX reconstructed the US images from FA. The PNN algorithm is the default algorithm as it is simple and fast. During the reconstruction of the US images into a volume, a voxel could already have a pixel value from previous US images. In this situation, the algorithm assigns the maximum pixel value from the allocated pixels in multiple US images. This removes black spots as shadows could occur easily during the US acquisition. The input variables for this PNN algorithm were a distance of 3 pixels and temporal calibration of −30 ms. Before analyzing the FA 3D volumes, the orientation of the volume had to be transformed as it was not aligned in the form of a right-anterior–superior (RAS) coordinate system. The missing alignment is because there are no calibration marks on the EM sensors on how the sensors are related to the RAS coordinate system.

The US images from MA were stacked into a 3D array. No reconstruction algorithm is required as the acquisition is performed while moving in only one direction along the rails, and the motor controls the step size.

### Evaluation criteria

Quantifying the 3D image quality of the US volume was performed using the following metrics: contrast resolution, axial and elevation resolution, axial and elevation distance calibration, stability, inter-operator variability, and intra-operator variability. The Guidelines for Quality Control Radiological Machines by the Dutch Association of Clinical Physics recommend these metrics as they are most common for image quality assessment [20].

**Fig. 2:**
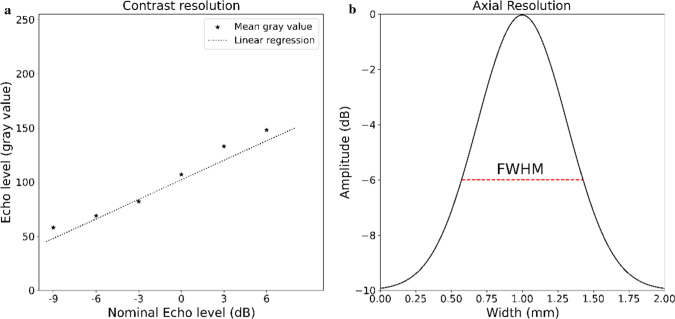
**a** (left): An example of mean gray values plotted against the nominal echo levels. The dotted line represents a linear regression of the gray values. The gradient of the linear regression is the contrast resolution. **b** (right): An example of how FWHM is measured. The peak of the curve represents a small marker. −6 dB is considered half the level of the maximum signal of the peak. The red striped line at −6 dB represents the full width of the peak (FWHM)

Contrast resolution is determined by measuring the average gray value in the five grayscale contrast cylinders and plotting those against the nominal value (−9, −6, −3, + 3, + 6 dB). The gradient of the linear regression line through these plots is the contrast resolution in gray value/dB, as shown in Fig. 2a.

**Fig. 3:**
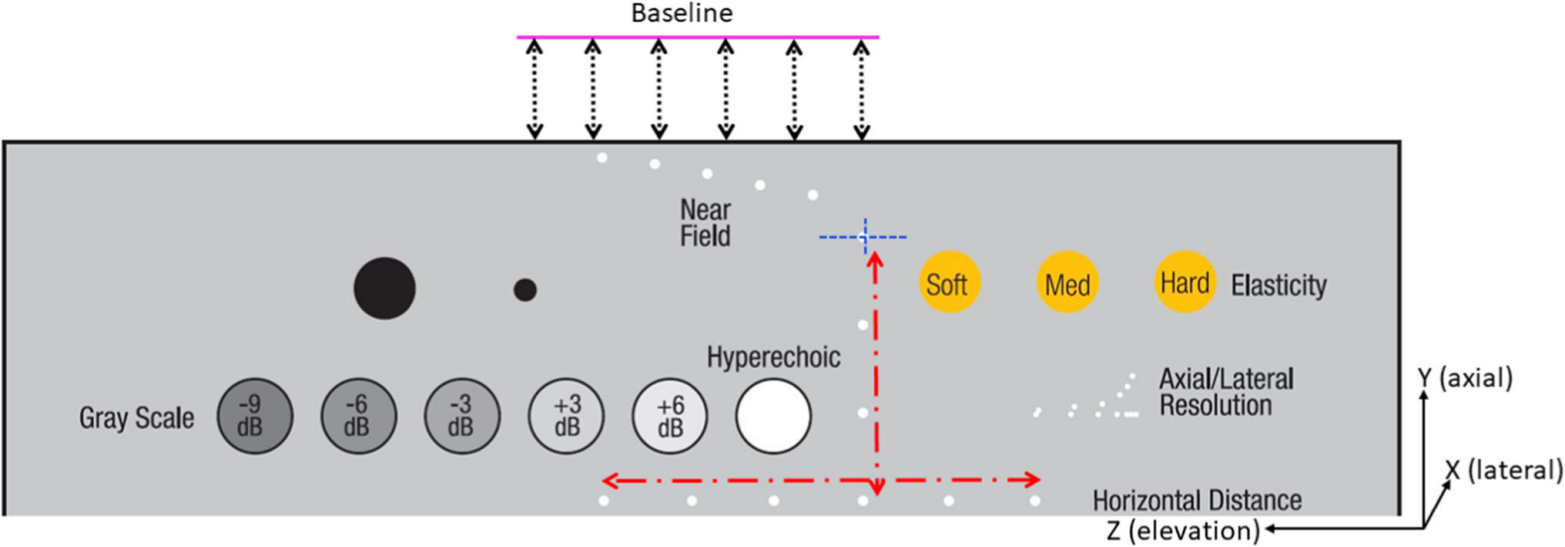
A schematic view of the phantom and its targets. The red long dash-dot arrows represent the distance calibration dots with a ten-mm spacing. The blue striped lines represent the resolution measure of a single marker. The black dotted arrows below the magenta baseline represent the distance measurement to the surface to determine the stability. The US transducer acquires images in the XY (axial-lateral) plane and moves in the Z (elevation) direction. See Appendix A for the full figure of the phantom datasheet

Additionally, the contrast resolution is used to determine the full width at half maximum (FWHM), which is the width of the peak at a −6 dB reduction of the maximum signal. The vertical distance marker at one cm depth (0,1 mm diameter) is used to draw a line profile in axial and elevation directions, shown as the dashed blue line in Fig. 3. The pixel intensities along the line were extracted to measure the FWHM. Fig. 2b shows an example of measuring the FWHM.

Distance calibration in axial and elevation directions is measured by manually selecting the two pixels with the highest gray value representing the distance markers, shown as the red long dash-dot arrows in Fig. 3. The distance is measured between two points, which are separated as far as possible. If multiple pixels have the same highest gray value, the center pixel was selected. The distance error is the absolute difference between the measured distance $D_{m}$ and the actual distance $D_{a}$ of the markers, as shown in Eq. 1. The actual distance in the axial direction is 30 mm and in the elevation direction is 50 mm.

1 $$ {\text{Distance error}} = \left| {D_{a} - D_{m} } \right| $$

**Fig. 4:**
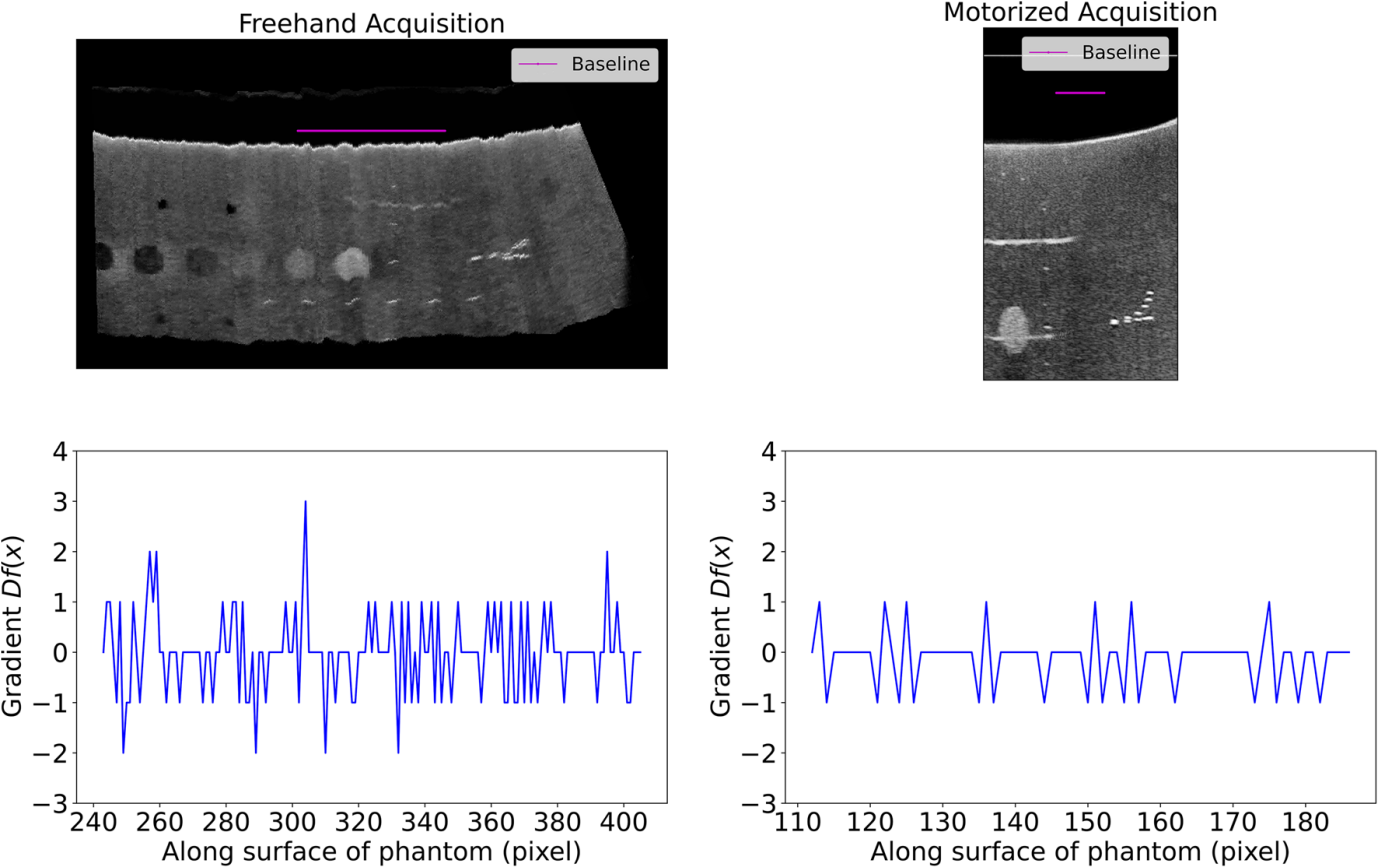
Upper: An example of the ZY plane of a 3D US volume with the baseline in magenta of FA (left) and MA (right). Lower: A plotted representation of the gradient of the phantom's surface. Because the cable from the US system is horizontally attached to the transducer and the water well is attached to the phantom, acquiring an image in the XY plane is limited for the moving range. This moving range was larger with FA as the operator could adjusted the angle of the transducer not to be perpendicular to the phantom so the cable was less blocked by the water well

The stability is assessed by quantifying the image surface’s smoothness of the phantom after 3D reconstruction. First, the middle YZ plane of the US volume is selected and then converted into a binary image using a threshold of gray value 100, and the phantom’s surface is segmented. Secondly, a baseline was created with a fixed location above the phantom’s surface and an elevation length of 25% of the 3D volume, shown as the magenta line in Figs. 3 and 4. From each pixel ($x_{1} ,x_{2} , \ldots ,x_{n}$) along the baseline, the perpendicular distance ($f\left( x \right)$) to the phantom’s surface was measured in pixels. The gradient of $f\left( x \right)$ was assessed by computing the derivative ($Df\left( x \right)$). As the phantom’s surface is slightly curved, a constant $Df$ of 0 will not occur. Therefore, the root-mean-squared (${\text{RMS}}$) average of distance deviations is computed by Eq. 2, including the minimum ($\min \left( {{\text{Df}}} \right)$) and maximum ($\max \left( {{\text{Df}}} \right)$) deviations. In other words, although one can expect a deviation of ${\text{RMS}}$ in the next slice in the 3D volume, the deviation could be $\min \left( {{\text{Df}}} \right)$ or $\max \left( {{\text{Df}}} \right)$ and appear consecutively in the successive slices. Multiplying the $\min \left( {{\text{Df}}} \right)$, $\max \left( {{\text{Df}}} \right)$, and ${\text{RMS}}$ by the axial pixel spacing converts all measures into mm.

2 $$ \begin{array}{*{20}c} {{\text{RMS}} = \sqrt {\frac{1}{n}\left( {{\text{Df}}\left( {x_{1} ,x_{2} , \ldots ,x_{n} } \right)} \right)^{2} } } \\ \end{array} $$

A linear mixed model determined statistical differences between FA and MA. Differences were considered significant by *p*-values < 0.05. Paired-samples *t*-test determined the statistical differences between stepwise and continuous movement within MA, and between with and without compound imaging for stepwise movement with a significance level of *α* = 0.05.

The intra-operator variability was measured among three repetitive acquisitions of each combination of experimental settings. A second operator (B) performed three acquisitions for FA and three in the MA experiments for both movements but with a step size of 0.5 mm to determine the inter-operator variability. Intra-class correlation coefficient (ICC) determined the statistical agreement between the two operators with a significance level of *α* = 0.05.

### Experiments

Table 1 shows all the variables for each experiment. All experiments were performed at the 0.5 dB side of the phantom. During each experiment, the operator completed three acquisitions. Only operator A acquired with compound imaging.

## Results

In this study, 3D US acquisitions were performed in a freehand or motorized fashion with movement only in the elevation direction (Z, Fig. 3). Because the cable from the US system is horizontally attached to the transducer and the water well is attached to the phantom, acquiring an image in the XY plane is limited for the moving range, and in the ZY plane was entirely not possible. The moving range was larger with FA as the operator could adjust the angle of the transducer not to be perpendicular so the cable was less blocked by the water well. Depending on the target in the phantom, the depth of the field of view ranged between five and six cm. The FA, including reconstruction, took on average (SD) 32.3 (± 3.2) seconds versus a range of 9–448 s with MA, which depends on the input variables. The FA resulted in 3D volumes of mean dimensions 441 × 375 × 632 with an isotropic voxel spacing of approximately 0.22 × 0.22 × 0.22 mm. MA obtained 3D volumes of 668 × 544 × *Z*, with *Z* being step size multiplied by the input variable distance, ranging from 60 to 600 acquired images. The pixel spacing in the axial and lateral directions is equal and ranges between approximately 0.10–0.12 mm, depending on the set depth of the US system. The pixel spacing in the elevation direction was the input variable step size, ranging from 0.1 to 1 mm.

Table 2 shows the mean (SD) of both FA and MA for all metrics except variability. The metrics resolution in the elevation direction, distance calibration in the axial direction, and stability show statistically significant differences between FA and MA. FA results in the lowest FWHM (0.55 (± 0.20) mm) in elevation resolution compared to MA (1.02 (± 0.44) mm) (*p* = 0.003). For distance calibration error in the axial direction, MA provides the lowest error (1.79 (± 0.07) mm) compared to FA (2.27 (± 0.40) mm) (*p* < 0.0001). Additionally for stability, MA results in a lower RMS (0.06 (± 0.01) mm) compared to FA (0.17 (± 0.01) mm) (*p* < 0.0001).

**Table 2:** The results of FA and MA per evaluation metric. The freehand method includes six acquisitions. The motorized method includes 33 acquisitions. The statistically significant differences between FA and MA are presented in bold

Metric	Freehand acquisition Mean (± standard deviation)	Motorized acquisition Mean (± standard deviation)	*p*-value (*α* = 0.05)
Contrast resolution	6.2 (± 2.1) gray value/dB	6.3 (± 0.3) gray value/dB	*p* = 0.486
Resolution axial	0.51 (± 0.26) mm	0.33 (± 0.08) mm	*p* = 0.209
Resolution elevation	0.55 (± 0.20) mm	1.02 (± 0.44) mm	***p*** = ** 0.003**
Distance calibration error axial	2.27 (± 0.40) mm	1.79 (± 0.07) mm	***p* < 0.0001**
Distance calibration error elevation	4.22 (± 4.75) mm	2.00 (± 0.81) mm	*p* = 0.211
Stability	0.17 (± 0.01) mm Min; Max: −0.68; 0.68 mm	0.06 (± 0.01) mm Min; Max: −0.20; 0.20 mm	***p* < 0.0001**

Detailed information about the influence of the variables of MA on the image quality can be found in Appendix B. Table 3 shows the results of the variability between the operators A and B. The agreement between operators A and B is only statistically significant for MA with stepwise movement for the metric contrast resolution. Table 3 shows that in general for each metric MA results in lower standard deviations within and between the operators than FA.

**Table 3:** The results of operators A and B for both FA and MA for all evaluation metrics representing the variability. The three measurements per combination of the method, movement and operator variables (step size 0.5 mm for MA) are included in the intraclass correlation coefficient (ICC). Statistical significant agreement (α=0.05) between operators A and B are presented in bold

Variability					
Metric	Method	Movement	Operator A Mean (± standard deviation)	Operator B Mean (± standard deviation)	ICC (*p*-value, *α* = 0.05)
Contrast resolution (gray value/dB)	Freehand	Freehand	6.91 (± 2.73)	5.46 (± 0.18)	ICC = −0.066 (*p* = 0.528)
	Motorized	Stepwise	6.25 (± 0.14)	6.10 (± 0.11)	**ICC = 0.677 (*p* = 0.011)**
		Continuous	6.64 (± 0.08)	6.62 (± 0.03)	ICC = −0.792 (*p* = 0.715)
Resolution axial (mm)	Freehand	Freehand	0.58 (± 0.36)	0.44 (± 0.04)	ICC = −0.240 (*p* = 0.586)
	Motorized	Stepwise	0.21 (± 0.00)	0.37 (± 0.02)	ICC = 0.004 (*p* = 0.420)
		Continuous	0.33 (± 0.06)	0.34 (± 0.02)	ICC = 0.179 (*p* = 0.435)
Resolution elevation (mm)	Freehand	Freehand	0.70 (± 0.18)	0.40 (± 0.03)	ICC = 0.078 (*p* = 0.403)
	Motorized	Stepwise	0.96 (± 0.09)	0.57 (± 0.05)	ICC = −0.008 (*p* = 0.539)
		Continuous	1.04 (± 0.03)	1.12 (± 0.05)	ICC = −0.391 (*p* = 0.829)
Distance Calibration axial (mm)	Freehand	Freehand	2.09 (± 0.34)	2.46 (± 0.36)	ICC = 0.614 (*p* = 0.102)
	Motorized	Stepwise	1.83 (± 0.00)	1.83 (± 0.02)	ICC = 0.00 (*p* = 0.500)
		Continuous	1.72 (± 0.01)	1.72 (± 0.01)	ICC = 0.500 (*p* = 0.300)
Distance calibration elevation (mm)	Freehand	Freehand	6.39 (± 5.93)	2.06 (± 0.75)	ICC = 0.003 (*p* = 0.498)
	Motorized	Stepwise	1.5 (± 0.00)	1.52 (± 0.70)	ICC = 0.00 (*p* = 0.500)
		Continuous	3.02 (± 0.21)	2.55 (± 0.00)	ICC = 0.00 (*p* = 0.500)
Stability (mm)	Freehand	Freehand	0.18 (± 0.00)	0.17 (± 0.01)	ICC = −0.720 (*p* = 0.725)
	Motorized	Stepwise	0.06 (± 0.00)	0.06 (± 0.00)	ICC = −0.589 (*p* = 0.825)
		Continuous	0.06 (± 0.00)	0.06 (± 0.00)	ICC = 0.00 (*p* = 0.500)

## Discussion

This study compared and evaluated the image quality of 3D US volume between FA and MA following the metrics: contrast resolution, resolution, distance calibration, stability, and variability. The main finding is that MA resulted in better image quality based on lower distance calibration error in the axial direction, more stability, and less variability. These results suggest acquiring 3D US volumes for intra-operative *ex vivo* margin assessment in a motorized fashion. To our knowledge, this study describes the first comparison between freehand and motorized 3D US.

Most studies report improved inter-observer variability with motorized systems, similar to our study. Jiang et al. report that US acquisitions with a robotic-arm (KUKA LBR iiwa) can overcome the inter-observer variability [21]. Furthermore, Kojcev et al. compare thyroid size measurements between 3D US with a robotic-arm (KUKA LBR iiwa) and manually operated 2D US, showing that the 3D US with a robotic-arm leads to more consistent and reproducible measurements [22]. Although the difference between MA and FA of 3D US remains unclear, the operator-dependent FA method could explain the lower reproducibility.

One of the strengths of our study is that we evaluated this operator dependence between the two acquisition methods and the two operators. The results of this evaluation show that measurements on a 3D volume by MA are reproducible and not operator-dependent. These results are interpreted from a sample size of *n* = 9. Additionally, the inter-observer variability is evaluated for two operators. These choices are based on the preliminary results showing very low variance among measurements. Extending the number of acquisitions or operators would probably not change these outcomes. No statistically significant agreement was found between the observers assessed by the ICC because of the small sample size.

A second vital aspect of this phantom study is the simulation of the clinical setting by adding water as a medium to transmit high-frequency sound waves. Secondly, water ensures that no pressure is applied to the phantom to prevent deformation. Moreover, the operator could not use the phantom’s surface as a guide to stabilize their hand during EM-navigated FA. Although the phantom is of different size and consists of targets in different shapes and echogenicity compared to ex vivo tongue specimens, this phantom suits very well for assessing the image quality of 3D US volumes. As tongue specimens are of smaller size with a minimal depth, we only used the imaging targets at the phantom’s upper layers. This study successfully mimicked the essential aspects of the clinical setup used during margin assessment.

This study was limited to only one probe-US system combination. Increasing the number of combinations would lead to more variables and distract from the study’s aim to compare and evaluate the image quality of 3D US volumes acquired between the freehand and motorized fashion. The various measurements of different combinations of probes and US systems should be considered, as Sassaroli et al. [23] report.

Finally, other studies about image quality use the evaluation metrics as applied in this study as well [23]. Guidelines suggest that annual inspections of probes and US systems should be evaluated following these metrics [20]. The use of the selected metrics facilitates the comparison between studies about the image quality of 3D US.

The main differences between the two acquisition methods are the way the transducer moves along the target and the type of method for reconstructing 2D US images into a 3D US volume. FA requires an EM field in which the EM sensors continuously track the position and orientation of the US transducer. One drawback of this technique is that the presence of ferromagnetic materials interferes with the EM field and thus negatively influences the tracking accuracy of the EM system up to several millimeters. During our experiment, we carefully prevented any interference with ferromagnetic materials by replacing everything with plastic alternatives, but we cannot guarantee no interference occurred during the experiments. It was expected that motorized movement is more stable and consistent than a human hand, showing differences in the metrics of stability and variability. In the same way, stacking the 2D images was thought to result in higher resolution as no interpolation would blur the target’s edges.

No significant difference in contrast resolution was observed between FA and MA. This absence of difference can be explained by the fact that contrast resolution was measured by taking the mean pixel intensity in a much larger area than the three pixels distance of the PNN algorithm. Thus, this algorithm could not affect the contrast resolution. Moreover, the pixel intensities are similar for both methods, as the probe-US system combination is equal.

Comparing the resolution’s results showed that FA has a statistically significant lower elevation’s FWHM than MA. The PNN algorithm could cause this difference in resolution. While moving the probe along the phantom, the marker is visible in multiple images, while the marker has a size of 0.1 mm. Therefore, it can be concluded that the slice thickness at the focus depth of the US images is more than 0.1 mm. Stacking those images into a 3D US volume with a fixed step size results in a marker with a stretched-out representation. The PNN algorithm compensates for this mechanism due to the interpolation; therefore, FA provides the lowest FWHM in the elevation direction of the two methods at the focus depth. In Fig. 4, the deeper resolution markers are more horizontally stretch-out than the superficial marker at one-cm depth, which is used for the measurement of the FWHM. Based on visual inspection of Fig. 4 only, one would conclude that MA has a lower elevation’s FWHM for the deeper markers. We chose to measure the FWHM at the top marker and not the lower markers because in our clinical setup, the tumor is more superficial, and therefore, superficial markers are of higher interest.

MA performs the lowest error in axial distance calibration. During FA, the probe is not vertically aligned with the phantom’s vertical calibration markers due to the manual orientation of the probe. As a result, not all markers are represented in the same 2D US images. Again, FA results in deviations in the probe’s height, which the PNN algorithm cannot entirely compensate for. This means that similar height deviations can be seen in the pixels representing the markers, causing larger errors in distance calibration in the axial direction. Although other reconstruction algorithms from CustusX were not investigated, we do not expect that a different algorithm could compensate for these large errors. Future research may investigate the influence of reconstruction algorithms for FA separately. A limitation of the metric distance calibration for both directions is the manual selection of the pixel with the highest value. Distance measurements were computed by the same person performing the acquisition by selecting a coordinate within a pixel. The axial pixel size for FA was 0.22 mm and for MA 0.12 mm. This results in a manual selection error within a range of twice the pixel size for both acquisition methods.

The stability, measured by the RMS, was significantly better with MA. The RMS means that in the next slice, it can be expected that the same target will be represented by pixels with a mean axial deviation of 0.06 mm in the case of MA and within a range of −0.20 and 0.20 mm. On the contrary, FA resulted in a mean axial deviation of 0.17 mm and ranged to ± 0.68 mm. This deviation is a quantitative measure of manual instability during the acquisition. Considering that these deviations could occur in two consecutive slices, as shown in Fig. 4, a deviation of 1.3 mm would cause severe interference when measuring distances such as the resection margin. As the guidelines report an adequate margin of > 5 mm [1], the instability of FA could result in a deviation in the margin of over 20%.

Based on the higher stability and lower intra-operative variability of MA, we would advise acquiring 3D US volume with MA. Although the stepwise movement has a statistically significant lower distance calibration error in the elevation direction, we prefer a continuous movement with a step size of 0.5 mm as time is a more important variable for us.

No statistically significant agreement between operators A and B was seen, except for the contrast resolution for MA with stepwise movement. Due to the insignificance of the other metrics, conclusions about the inter-operator variability assessed by the ICC could not be derived. The standard deviations within operators A or B are generally higher with FA than MA, indicating a higher intra-operator variability. According to the known variability of manual performance, manual instability is likely the leading cause of the variability in our study.

Our study contributes to improving the intra-operative margin assessment of oncological specimens. Without the need for an experienced operator, motorized 3D US enables reproducible, consistent, and automatic acquisition of entire resected oncological specimens resulting in reliable measurements. MA requires fewer devices than FA and is, therefore, more accessible for implementation in smaller operating rooms. Once implemented, we advise using MA with a continuous movement with 0.5 step size, as assessment time should be as limited as possible. From the financial point of view, MA is more favorable as well. Various motorized systems are available for 1/10^th^ of the costs of an EM tracking system.

In conclusion, MA is more accurate in axial distance measurement and more stable than FA, whereas FA has the lowest resolution in the elevation direction. The very low variability of MA ensures that measurements are reproducible, consistent, and not operator-dependent. Therefore, MA is the preferred option for 3D US in the context of intra-operative *ex vivo* margin assessment. The next step is to clinically validate the motorized 3D US system by investigating the agreement of clinical measurement between motorized 3D US and histopathology. Additionally, we are now developing a deep learning model to automatically perform the intra-operative margin measurements in the motorized 3D US volume, as manual segmentation is time-consuming. The results will be reported in subsequent publications.

## Data Availability

Research data are available upon reasonable request. See corresponding authors for contact details.

## Appendix A

Datasheet of the CIRS multi-purpose phantom.

## Appendix B

The influence of the variables within motorized acquisition on the 3D image quality.

Table 4 in Appendix B shows the results for all metrics except variability between the stepwise and continuous movement of MA, including acquisition and reconstruction time. Statistical significant differences are present in the following metrics: contrast resolution is higher for continuous movement at step size: 0.5–0.2–0.1 mm (*p* < 0.05); resolution in axial direction is lower for stepwise movement at step size 1 mm (*p*  = 0.015); resolution in elevation direction is lower for stepwise movement at step size 1–0.5 mm (*p* < 0.05); distance calibration error in axial direction is lower for continuous movement at step size 1–0.5 mm (*p* < 0.01); distance calibration error in elevation direction is lower for stepwise movement at all step size (*p* < 0.005); stability is lower for continuous movement at step size 0.2–0.1 mm (*p* < 0.05); and time is lower for continuous movement at all step size (*p* < 0.005).

**Table 4:** The results of stepwise and continuous movement of MA for all evaluation metrics including time. For the step size 1mm-0.2mm-0.1mm three measurements for both stepwise and continuous are included in the paired-samples t-test. For the 0.5mm step size operators A and B are both included so six measurements for both stepwise and continuous are included in the paired-samples t-test. Statistical significant differences between stepwise and continuous are presented in bold

Motorized acquisition			
Step size	Stepwise Mean (± standard deviation)	Continuous Mean (± standard deviation)	*p*-value (*α* = 0.05)
*Contrast resolution (gray value/dB)*			
1 mm	6.2 (± 0.03)	6.3 (± 0.13)	*p* = 0.4567
0.5 mm	6.2 (± 0.14)	6.6 (± 0.06)	***p***** = 0.0005**
0.2 mm	6.0 (± 0.07)	6.8 (± 0.07)	***p***** = 0.0135**
0.1 mm	5.9 (± 0.05)	6.4 (± 0.03)	***p***** = 0.0006**
*Resolution axial (mm)*			
1 mm	0.26 (± 0.01)	0.49 (± 0.03)	***p***** = 0.0150**
0.5 mm	0.29 (± 0.08)	0.34 (± 0.06)	*p* = 0.3653
0.2 mm	0.33 (± 0.01)	0.34 (± 0.07)	*p* = 0.0501
0.1 mm	0.29 (± 0.02)	0.27 (± 0.01)	*p* = 0.2693
*Resolution elevation (mm)*			
1 mm	0.75 (± 0.03)	2.23 (± 0.41)	***p***** = 0.0356**
0.5 mm	0.76 (± 0.21)	1.08 (± 0.06)	***p***** = 0.0334**
0.2 mm	0.92 (± 0.07)	1.07 (± 0.01)	*p* = 0.1151
0.1 mm	0.90 (± 0.06)	0.97 (± 0.04)	*p* = 0.2506
*Distance calibration error axial (mm)*			
1 mm	1.83 (± 0.00)	1.64 (± 0.03)	***p***** = 0.0097**
0.5 mm	1.83 (± 0.02)	1.72 (± 0.01)	***p***** = 0.0000**
0.2 mm	1.79 (± 0.05)	1.71 (± 0.00)	*p* = 0.1749
0.1 mm	1.86 (± 0.00)	1.85 (± 0.00)	*p* = 0.0572
*Distance calibration error elevation (mm)*			
1 mm	1.36 (± 0.43)	3.39 (± 0.49)	***p***** = 0.0003**
0.5 mm	1.51 (± 0.50)	2.79 (± 0.28)	***p***** = 0.0041**
0.2 mm	1.42 (± 0.17)	2.71 (± 0.08)	***p***** = 0.0024**
0.1 mm	1.42 (± 0.09)	2.00 (± 0.03)	***p***** = 0.0048**
*Stability (mm)*			
1 mm	0.08 (± 0.00)	0.08 (± 0.00)	*p* = 0.4226
0.5 mm	0.06 (± 0.00)	0.07 (± 0.0)	*p* = 0.8255
0.2 mm	0.06 (± 0.01)	0.05 (± 0.00)	***p***** = 0.0269**
0.1 mm	0.05 (± 0.00)	0.04 (± 0.00)	***p***** = 0.0293**
*Time to acquire and reconstruct (seconds)*			
1 mm	32.5 (± 0.0)	7.5 (± 2.4)	***p***** = 0.0046**
0.5 mm	68.2 (± 1.2)	14.6 (± 2.3)	***p***** = 0.0000**
0.2 mm	188.7 (± 0.2)	53.8 (± 0.1)	***p***** = 0.0000**
0.1 mm	448.1 (± 4.0)	171.1 (± 1.8)	***p***** = 0.0002**

For the metrics in the elevation direction, it is important to take into account the elevation pixel spacing of FA is 0.2 mm, while for MA this elevation pixel spacing is, depending on the experiment, 0.1–1 mm. The step size of 0.5 and 1 mm limits the ability to reach similar elevation pixel spacing as FA. But, Table 4 shows that a step size of 0.1 and 0.2 mm still results in similar resolutions in the elevation direction. Thus, comparing FA with only the step sizes of 0.1 and 0.2 mm of MA results in the same conclusion: FA has a lower resolution in the elevation direction. For the distance calibration error in the elevation direction, MA shows lower distance errors for the step size of 0.1 and 2.0 mm. Taking into account only the step size of 0.1 and 0.2 mm when comparing MA to FA, this might result in a lower distance error of MA of statistical significance.

Table 5 in Appendix B presents the different results of the variable compound imaging during stepwise MA. Applying compound imaging results in statistically significant lower resolution in elevation direction (*p*  = 0.0211) and lower distance calibration in elevation direction (*p*  = 0.0379). On the other hand, not applying compound imaging results in statistically significant lower resolution in axial direction (*p*  = 0.0267) and lower distance calibration error in axial direction (*p*  = 0.0000).

**Table 5:** The results of applying compound imaging during MA with step size 0.5 mm stepwise movement for all metrics. For each metric, three measurements of operator A without and three measurements of operator A with compound imaging are included in the paired-samples t-test. Statistical significant differences between compound imaging and not compound imaging are presented in bold

Motorized acquisition—stepwise movement–0.5 mm step size			
Compounding	No Mean (± standard deviation)	Yes Mean (± standard deviation)	*p*-value (*α* = 0.05)
Contrast resolution (gray value/dB)	6.24 (± 0.14)	6.03 (± 0.13)	*p* = 0.3549
Resolution axial (mm)	0.21 (± 0.00)	0.30 (± 0.02)	***p***** = 0.0267**
Resolution elevation (mm)	0.96 (± 0.09)	0.67 (± 0.07)	***p***** = 0.0211**
Distance calibration error axial (mm)	1.83 (± 0.00)	1.86 (± 0.00)	***p***** = 0.0000**
Distance calibration error elevation (mm)	1.5 (± 0.00)	1.1 (± 0.11)	***p***** = 0.0379**
Stability (mm)	0.05 (± 0.00))	0.05 (± 0.00)	*p* = 0.4226
